# Pneumonectomy for primary lung cancer: contemporary outcomes, risk factors and model validation

**DOI:** 10.1093/icvts/ivab340

**Published:** 2021-12-06

**Authors:** Annemarie Brunswicker, Marcus Taylor, Stuart W Grant, Udo Abah, Matthew Smith, Michael Shackcloth, Felice Granato, Rajesh Shah, Kandadai Rammohan, the North West Thoracic Surgery Collaborative (NWTSC)†, Leah Argus, Sarah Michael, Sabrina Mason, Dilraj Bhullar, Emmanuel Obale, NilsCristopher Fritsch

**Affiliations:** 1 Department of Cardiothoracic Surgery, Manchester University Hospital NHS Foundation Trust, Wythenshawe Hospital, Manchester, UK; 2 Division of Cardiovascular Sciences, University of Manchester, ERC, Manchester University Hospital NHS Foundation Trust, Manchester, UK; 3 Department of Cardiothoracic Surgery, Liverpool Heart & Chest Hospital, Liverpool, UK

**Keywords:** Pneumonectomy, Non-small-cell lung cancer, Risk model, 90-Day mortality

## Abstract

**OBJECTIVES:**

Despite the increased rate of adverse outcomes compared to lobectomy, for selected patients with lung cancer, pneumonectomy is considered the optimal treatment option. The objective of this study was to identify risk factors for mortality in patients undergoing pneumonectomy for primary lung cancer.

**METHODS:**

Data from all patients undergoing pneumonectomy for primary lung cancer at 2 large thoracic surgical centres between 2012 and 2018 were analysed. Multivariable logistic and Cox regression analyses were used to identify risk factors associated with 90-day and 1-year mortality and reduced long-term survival, respectively.

**RESULTS:**

The study included 256 patients. The mean age was 65.2 (standard deviation 9.4) years. In-hospital, 90-day and 1-year mortality were 6.3% (*n* = 16), 9.8% (*n* = 25) and 28.1% (*n* = 72), respectively. The median follow-up time was 31.5 months (interquartile range 9–58 months). Patients who underwent neoadjuvant therapy had a significantly increased risk of 90-day [odds ratio 6.451, 95% confidence interval (CI) 1.867–22.291, *P* = 0.003] and 1-year mortality (odds ratio 2.454, 95% CI 1.079–7.185, *P* = 0.044). Higher Performance Status score was associated with higher 1-year mortality (odds ratio 2.055, 95% CI 1.248–3.386, *P* = 0.005) and reduced overall survival (hazard ratio 1.449, 95% CI 1.086–1.934, *P* = 0.012). Advanced (stage III/IV) disease was associated with reduced overall survival (hazard ratio 1.433, 95% CI 1.019–2.016, *P* = 0.039). Validation of a pneumonectomy-specific risk model demonstrated inadequate model performance (area under the curve 0.54).

**CONCLUSIONS:**

Pneumonectomy remains associated with a high rate of perioperative mortality. Neoadjuvant chemoradiotherapy, Performance Status score and advanced disease emerged as the key variables associated with adverse outcomes after pneumonectomy in our cohort.

**Figure:**
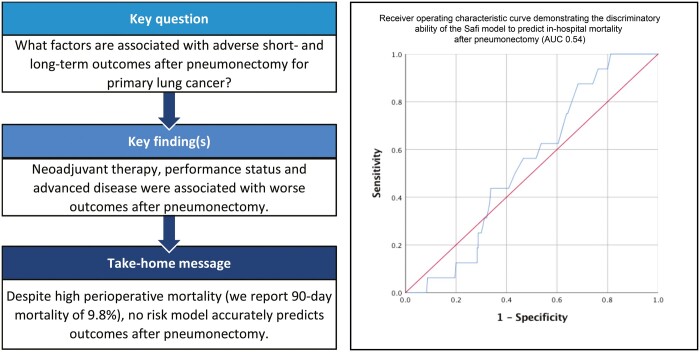

## INTRODUCTION

Lung cancer is the third most common cancer in the UK with over 47 000 new diagnoses each year [1]. Whilst the number of resections performed in the UK for primary lung cancer each year continues to rise, resection rates still vary widely across different regions of the country [2]. Overall, more than three-quarters of patients undergoing surgical resection for lung cancer will receive a lobectomy [2], and whilst there is increasing evidence that some lung cancers may be successfully treated with sublobar anatomical resections [3], a subgroup of patients may still require complex parenchymal-sparing procedures (such as sleeve lobectomy) or pneumonectomy to achieve complete resection.

Over recent years, the rate of complex parenchymal-sparing procedures has remained static but there has been a marked decline in the number of patients undergoing pneumonectomy [2]. Although outcomes have improved over time, patients undergoing pneumonectomy still have significantly higher rates of perioperative mortality compared to patients undergoing lobectomy or bilobectomy [2]. Nevertheless, despite this increased perioperative risk, for some patients with central or locally advanced tumours, pneumonectomy is often the only surgical treatment where complete resection can be achieved.

A number of studies have been undertaken aiming to identify risk factors associated with poor outcomes after pneumonectomy. Advanced age, poor pulmonary function tests and undergoing a right-sided operation have been associated with adverse outcomes after pneumonectomy [4–7]. Several clinical prediction models have been developed for perioperative mortality after thoracic surgery that include pneumonectomy as a risk factor [8]; however, only one model has been designed specifically for patients undergoing pneumonectomy [9]. No pneumonectomy-specific clinical prediction model for long-term survival has previously been developed.

The objective of this study was therefore to report contemporary outcomes for patients undergoing pneumonectomy for primary lung cancer and identify risk factors associated with 90-day mortality, 1-year mortality and worse overall survival.

## PATIENTS AND METHODS

### Ethics statement

Data were stored in the Northwest Clinical Outcomes Research Registry (NCORR) (IRAS 260294). The NCORR database has full ethical approval from the regional Research Ethics Committee of the Health Research Authority. This project was approved by the NCORR steering committee and individual patient consent was waived due to anonymization of the data prior to use and the retrospective nature of the project.

### Patients and data

All consecutive patients who underwent pneumonectomy for stages I-IV non-small-cell lung cancer at Manchester University NHS Foundation Trust and Liverpool Heart & Chest Hospital between January 2012 and December 2018 were included. All cases of non-small-cell lung cancer were confirmed pathologically. Staging was assigned based on the postoperative histological analysis according to the 7th edition of the Tumour Node Metastasis Classification for Lung Cancer. Patients undergoing completion pneumonectomy were not included in this series. The survival time was defined as the number of days from the date of surgery to the date of death. Of the thirteen surgeons who contributed patients to this series, eleven are dedicated thoracic surgeons, whilst 2 maintain a mixed cardiothoracic practice. Anaesthesia is provided by specialized cardiothoracic anaesthetists.

We have described our data collection methods in previous publications [10]. Variables missing >15% of data were excluded from analyses. For categorical variables, missing data was replaced with the most frequently occurring value. For continuous variables, missing data were replaced with either the mean (for normally distributed data) or median (for non-normally distributed data) value.

### Preoperative work-up

All patients underwent staging contrast computed tomography, positron emission tomography and pulmonary function tests (including full spirometry and gas transfer) as standard. Latterly, endobronchial ultrasound transbronchial needle aspiration, cranial magnetic resonance imaging, transthoracic echocardiogram and exercise testing (either shuttle walk test or cardiopulmonary exercise testing) were also undertaken routinely. A multidisciplinary team assessed each case prior to surgery and all patients were also discussed at specially convened high-risk multidisciplinary team meetings [11].

### Outcomes

The primary outcomes were 90-day mortality, 1-year mortality and overall survival. Based on recent publications highlighting that 90-day mortality is double 30-day mortality after lung resection, we feel that 90-day mortality is a superior measure of perioperative mortality and supersedes in-hospital mortality, 30-day mortality or any composite endpoint thereof [12]. Postoperative length of stay was assessed as a secondary outcome.

### Statistical analysis

Continuous variables are presented as mean ± standard deviation and median ± interquartile range (IQR) for normal and non-normally distributed variables, respectively. Discrete variables were presented as percentages. Due to the relatively low number of outcomes, available risk factors were rationalized for inclusion in all multivariable analyses. Risk factors were selected for inclusion based on clinical relevance. No evidence of collinearity between any covariates was identified (assessed using the Pearson correlation coefficient, the variance inflation factor and eigenvalues). The impact of variables on 90-day mortality and 1-year mortality was assessed using multivariable logistic regression. Adjusted odds ratios and 95% confidence intervals (CIs) were calculated. Multivariable Cox proportional hazards regression analysis was undertaken to identify factors independently associated with reduced survival. A combination of statistical and graphical measures was performed based on Schoenfeld residuals to check the proportional hazards assumption. Adjusted hazard ratios and 95% CIs were calculated. A survival curve was produced using the Kaplan–Meier method.

For model validation, we assessed model performance using measures of discrimination and calibration. Model discrimination was assessed by calculating the area under the receiver operating characteristic curve. Model calibration was assessed using flexible calibration plots and observed-to-expected (O:E) ratios. An O:E ratio above 1 represents that model systematically under-estimating risk (and vice versa).

All tests were two-sided and statistical significance was defined as *P*-value <0.05. All statistical analysis was undertaken using SPSS version 25 (SPSS, Inc., Chicago, IL, USA).

## RESULTS

### Patient characteristics

During the study period, 5029 patients underwent surgery for primary lung cancer at our centres, of whom 256 underwent pneumonectomy. A total of 320 complex lobectomies (defined as bilobectomy, sleeve resection or chest wall resection) and 3917 non-complex lobectomies were performed during the same period. Overall, 64.8% (*n* = 166) of patients underwent surgery at Manchester University NHS Foundation Trust, whilst the remaining 35.2% (*n* = 90) were operated on at Liverpool Heart and Chest Hospital. The majority of patients (57.4%, *n* = 147) were male and the mean age was 65.2 years (standard deviation 9.4 years). A minority (7.0%, *n* = 18) of patients undergoing pneumonectomy received neoadjuvant therapy (chemotherapy only, *n* = 6/18; radiotherapy only, *n* = 1/18; combined chemoradiotherapy, *n* = 11/18). For all patients where information regarding decision-making after neoadjuvant therapy was available, pneumonectomy was offered as a result of tumour downstaging. Most patients (68.0%, *n* = 174) underwent left pneumonectomy. Based on postoperative pathological staging, 12.5% (*n* = 32) of patients were classified as stage I, 45.7% (*n* = 117) were stage II and 41.8% (*n* = 107) were stage III/IV. The proportion of patients undergoing pneumonectomy for stage I disease did not change significantly over time [2012–2014: 12.4% (*n* = 14) vs 2015–2018: 12.6% (*n* = 32), *P* = 0.962]. Nodal disease was present in 64.1% (*n* = 164) of patients. The in-hospital mortality rate was 6.3% (*n* = 16) and the median postoperative length of stay was 6 days (IQR 5–8 days). Patient characteristics are summarized in Table 1.

**Table 1:** Patient characteristics

Variable	Number	Missing data (%)
Age, years (mean ± SD)	65.2 years (9.4)	0
<55	12.5% (*n* = 32)	
55–65	35.5% (*n* = 91)	
>65	52.0% (*n* = 133)	
Male	57.4% (*n* = 147)	0
History of cancer	23.0% (*n* = 59)	3.9
Neoadjuvant therapy	7.0% (*n* = 18)	11.7
Chemotherapy	2.3% (*n* = 6)	
Radiotherapy	0.4% (*n* = 1)	
Chemoradiotherapy	4.3% (*n* = 11)	
Palliative indication for surgery	0.0% (*n* = 0)	0
Raised preoperative leucocyte count	16.0% (*n* = 41)	1.2
ASA ≥3	51.6% (*n* = 132)	0.4
PS ≥2	38.3% (*n* = 98)	2.3
NYHA ≥3	5.9% (*n* = 15)	2.0
% predicted FEV1 (mean ± SD)	82.14% (18.80%)	6.3
% predicted FVC (mean ± SD)	97.86% (18.31%)	7.4
% predicted DLCO (mean ± SD)	71.13% (16.67%)	9.8
Creatinine (mean ± SD)	74.9 (16.7)	9.4
Current alcohol use	9.0% (*n* = 23)	9.0
Anaemia	34.9% (*n* = 89)	10.9
Hypercholesterolaemia	9.8% (*n* = 25)	2.0
Hypertension	28.1% (*n* = 72)	2.0
Smoking	87.5% (*n* = 224)	2.0
Coronary artery disease	7.4% (*n* = 19)	10.2
COPD	35.9% (*n* = 92)	2.7
Cerebrovascular disease	3.9% (*n* = 10)	3.9
Right-sided resection	32.0% (*n* = 82)	0
TNM stage		0
Stage I	12.5% (*n* = 32)	
Stage II	45.7% (*n* = 117)	
Stage III/IV	41.8% (*n* = 107)	
Nodal status		0
N0	35.9% (*n* = 92)	
N1	42.6% (*n* = 109)	
N2	21.5% (*n* = 55)	
Postoperative histology		
Adenocarcinoma	25.4% (*n* = 65)	0
Squamous cell carcinoma	68.4% (*n* = 175)	0
Others	6.2% (*n* = 16)	0

ASA: American Society of Anaesthesiologists; COPD: chronic obstructive pulmonary disease; DLCO: diffusion capacity of the lung for carbon monoxide; FEV1: forced expiratory volume in 1 s; FVC: forced vital capacity; NYHA: New York Heart Association; PS: performance status; SD: standard deviation; TNM: tumour node metastasis classification.

### Ninety-day mortality

The 90-day mortality rate was 9.8% (*n* = 25). Univariable analyses are provided in the Supplementary Material. The 90-day mortality rates for patients who received neoadjuvant therapy (*n* = 18) versus those who did not (*n* = 238) were 33.3% (*n* = 6) and 8.0% (*n* = 19), respectively (*P* < 0.001). After adjustment using multivariable logistic regression, neoadjuvant therapy emerged as significantly associated with 90-day mortality. These results are shown in Table 2.

**Table 2:** Multivariable analysis for 90-day mortality

Variable	Odds ratio	95% confidence interval		*P*-value
Age	1.045	0.990	1.102	0.111
Male sex	1.982	0.746	5.267	0.170
Neoadjuvant therapy	6.451	1.867	22.291	0.003
PS	1.149	0.560	2.361	0.705
% predicted DLCO	0.989	0.961	1.018	0.466
Creatinine	0.995	0.968	1.023	0.741
Anaemia	0.861	0.343	2.165	0.751
Smoking	0.874	0.218	3.512	0.850
IHD	0.625	0.076	5.115	0.661
Right-sided resection	1.903	0.789	4.590	0.152
Advanced (stage III/IV) disease	0.444	0.164	1.203	0.111

DLCO: diffusion capacity of the lung for carbon monoxide; IHD: ischaemic heart disease; PS: performance status.

### One-year mortality

Observed 1-year mortality was 28.1% (*n* = 72) with 1-year outcome data available for 100% of patients. In total, 45.8% of these patients (*n* = 33/72) were known to have recurrent disease at the time of their death. Mortality at 1 year was higher for patients with advanced-stage (stage III/IV) disease in comparison to patients with early-stage (stage I/II) disease but this effect did not reach significance on univariable analysis (33.6% vs 24.2%, *P* = 0.096). Full details of the univariable analyses are available in the Supplementary Material. The 1-year mortality rates for patients who received neoadjuvant therapy (*n* = 18) versus those who did not (*n* = 238) were 50.0% (*n* = 9) and 26.5% (*n* = 63), respectively (*P* = 0.032). After adjustment using multivariable logistic regression, undergoing neoadjuvant therapy and higher Performance Status score were associated with significantly higher mortality at 1 year. Presence of advanced disease approached but did not meet the pre-defined threshold for statistical significance (odds ratio 1.769, 95% CI 0.989–3.164, *P* = 0.055). These results are shown in Table 3.

**Table 3:** Multivariable analysis for 1-year mortality

Variable	Odds ratio	95% confidence interval		*P*-value
Age	1.017	0.984	1.050	0.311
Male sex	0.947	0.509	1.760	0.863
Neoadjuvant therapy	2.454	1.079	7.185	0.044
PS	2.055	1.248	3.386	0.005
% predicted DLCO	1.001	0.983	1.019	0.952
Creatinine	1.003	0.984	1.021	0.772
Anaemia	1.805	0.989	3.293	0.054
Smoking	1.054	0.434	2.559	0.908
IHD	0.755	0.246	2.316	0.623
Right-sided resection	1.356	0.740	2.485	0.325
Advanced (stage III/IV) disease	1.769	0.989	3.164	0.055

DLCO: diffusion capacity of the lung for carbon monoxide; IHD: ischaemic heart disease; PS: performance status.

### Overall survival

**Figure 1:**
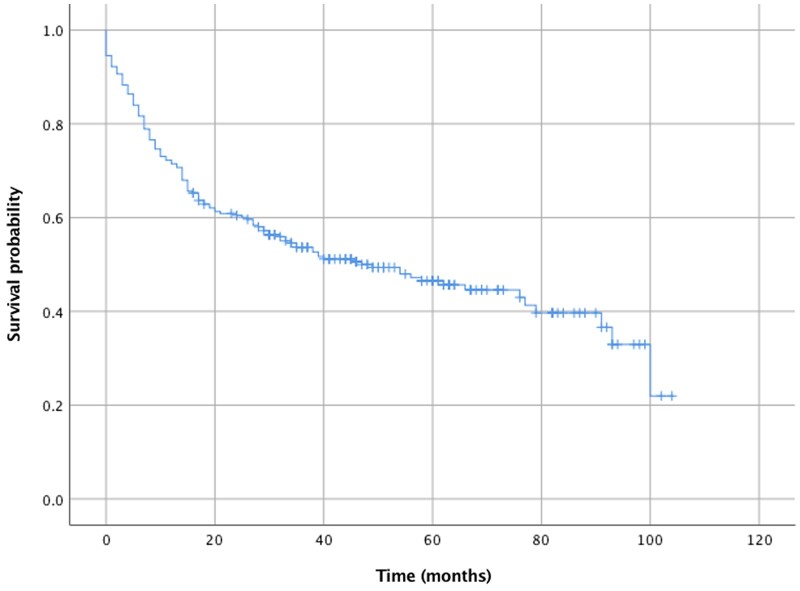
Kaplan–Meier curve for survival after pneumonectomy.

The median follow-up time was 31.5 months (IQR 9–58 months) and estimated median overall survival was 47 months (95% CI 27–67 months). Figure 1 shows the Kaplan–Meier curve for survival after pneumonectomy. The univariable analyses are available in the Supplementary Material. After adjustment, higher Performance Status score and undergoing surgery for advanced disease were associated with reduced overall survival. These results are shown in Table 4.

**Table 4:** Multivariable analysis for overall survival

Variable	Hazard ratio	95% confidence interval		*P*-value
Age	1.010	0.990	1.030	0.325
Male sex	0.948	0.650	1.382	0.780
Neoadjuvant therapy	1.369	0.733	2.556	0.325
PS	1.449	1.086	1.934	0.012
% predicted DLCO	1.000	0.989	1.011	0.982
Creatinine	1.003	0.992	1.014	0.581
Anaemia	1.176	0.807	1.713	0.399
Smoking	1.423	0.792	2.556	0.238
IHD	0.866	0.448	1.675	0.669
Right-sided resection	1.288	0.900	1.844	0.167
Advanced (stage III/IV) disease	1.433	1.019	2.016	0.039

DLCO: diffusion capacity of the lung for carbon monoxide; IHD: ischaemic heart disease; PS: performance status.

### Model validation

**Figure 2:**
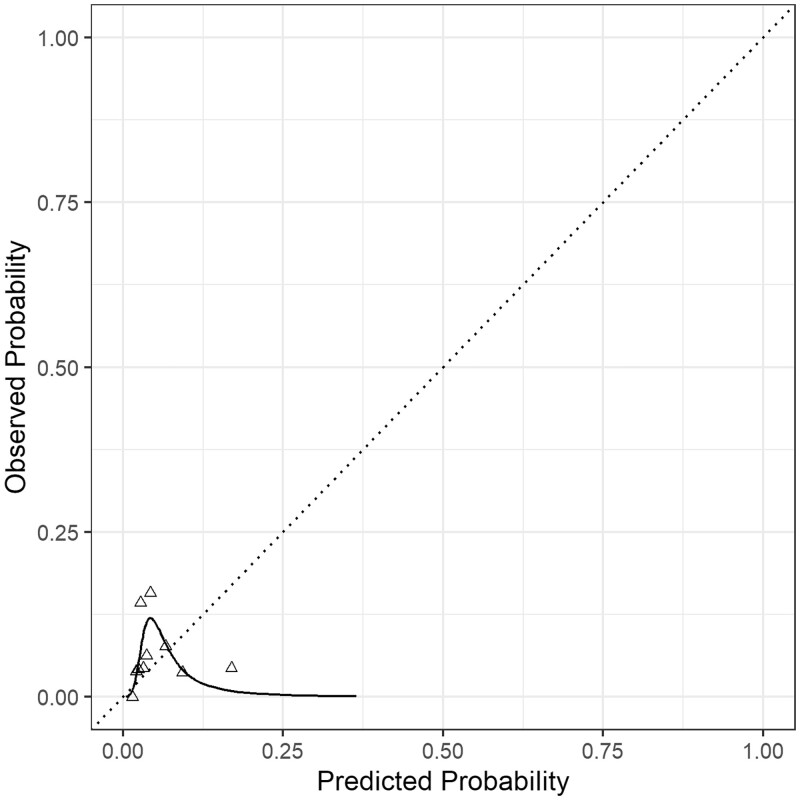
Calibration plot for the Safi model for predicting in-hospital mortality. The solid line is a flexible calibration curve fitted with restricted cubic splines. The triangle points are a binned calibration plot, with mean observed and expected risks calculated in 10 equally sized groups. The dotted line represents perfect calibration.

The model produced by Safi *et al.* developed specifically to predict in-hospital mortality for patients undergoing pneumonectomy was validated in our cohort of patients. Discrimination was poor, with an area under the receiver operating characteristic curve of 0.54. Although the overall O:E ratio (O:E ratio 1.23, *P* = 0.566) produced a result suggesting that calibration was acceptable, the flexible calibration plot, displayed in Fig. 2, does not reflect this finding and provides a visual representation of the extent of model miscalibration.

## DISCUSSION

This multicentre contemporary study of outcomes after pneumonectomy for primary lung cancer demonstrates in-hospital and 90-day mortality rates of 6.3% and 9.8%, respectively. These mortality rates are broadly similar to those published in other studies assessing outcomes after pneumonectomy [4, 5, 13]. Looking beyond the perioperative period, it is axiomatic that mid- and long-term outcomes for a cohort where over 40% of patients have advanced disease will be worse compared to the overall population of patients undergoing resection for lung cancer. Whilst the proportion of patients with stage I disease undergoing pneumonectomy may seem high (12.5%, *n* = 32), the figure is in keeping with other series [4, 14]. In our cohort, just under 30% of patients undergoing pneumonectomy had died within 1 year following surgery, just under half of whom (*n* = 33/72) had evidence of disease recurrence prior to death. Interestingly, although the 1-year mortality rate for patients with early disease was lower than for patients with advanced disease, this result did not reach significance, suggesting that mid-term mortality after pneumonectomy is likely to be multifactorial in origin, and should not be attributed to the presence of advanced malignant disease alone. However, it is important to note that although not statistically significant, there was a difference in mortality between the 2 groups of patients that is likely to have been important clinically. Moreover, the *P*-value associated with this result was approaching significance, suggesting that a greater sample size may have produced a result demonstrating a statistically significant difference in 1-year mortality between patients with early- and advanced-stage lung cancer.

Identifying patients at an increased risk of mortality after thoracic surgery is important. A number of risk prediction models have been developed to this end and these models include over 50 different predictors [8]. Often predictors that are strongly associated with mortality in some studies are found to have no impact on mortality in others. This heterogeneity is also seen in the studies where a multivariable analysis has been undertaken to identify predictors associated with postoperative mortality after pneumonectomy [5, 7, 15–18]. Despite representing a small group (*n* = 18), in this cohort neoadjuvant therapy was significantly associated with higher 90-day and 1-year mortality. The deleterious effect of neoadjuvant chemoradiotherapy on outcomes after pneumonectomy has also been previously demonstrated [19, 20], although we were unable to find any previous studies where this significance was retained after multivariable adjustment.

Undergoing a right-sided resection did not emerge as significant in any of the multivariable analyses conducted in this study. Despite not reaching significance on univariable analysis either, a clinically significant higher rate of mortality was seen for patients undergoing right-sided pneumonectomy, particularly at 90 days (14.6% vs 7.5%, *P* = 0.072). This finding correlates with analyses from other studies where the impact of laterality on both short-term outcomes and overall survival after pneumonectomy has been assessed [7, 17]. Indeed, despite not reaching statistical significance in this study, the relatively low *P*-value seen in the multivariable analysis for 90-day mortality (*P* = 0.152) suggests that a larger sample size may have led to this result reaching statistical significance.

Darling *et al.* also found a higher risk of bronchopleural fistula in patients undergoing a right-sided pneumonectomy [6]. This phenomenon can be explained by the fact that the right bronchial stump is more exposed, has blood supply from only one bronchial artery in the majority of cases and has less natural cover from mediastinal tissues. Bronchopleural fistula has itself also been found to be an independent predictor of poor outcomes after pneumonectomy, regardless of the laterality of the resection [5]. There are a number of other assumed pathophysiological mechanisms behind the increased risk of a right pneumonectomy including an increased right ventricular load delivering the entire cardiac output through the smaller remaining left lung and also loss of the larger right lung and the subsequent detrimental effect on respiratory function.

The fact that diffusion capacity of the lung for carbon monoxide (DLCO) did not emerge as significantly associated with adverse outcomes in this study is somewhat at odds with results from other contemporary studies looking at the role of pulmonary function tests in risk stratifying patients in the current era of thoracic surgery. For lung resection patients as a whole, the predictive role of spirometry values such as forced expiratory volume in 1 s has diminished over time [21] whilst DLCO has emerged as an increasingly important predictor of postoperative mortality and morbidity [22]. The dearth of studies looking solely at patients undergoing pneumonectomy where DLCO values are reported means that it is currently impossible to identify trends, although a subgroup of the multicentre UK Pneumonectomy Outcome Study where DLCO was reported found lower DLCO to be strongly associated with both 30-day mortality and the development of major postoperative complications [4].

The emergence of Performance Status score as being strongly predictive of long-term outcomes (significant association with higher 1-year mortality and reduced overall survival) but not short-term outcomes (no effect on 90-day mortality) after pneumonectomy suggests that preoperative baseline functional status in this cohort is of particular importance. Hence, additional attention should be paid to preoperative measures of physiological reserve such as the shuttle walk test and/or cardiopulmonary exercise test when attempting to determine the potential overall benefit which patients may gain from undergoing pneumonectomy. Performance Status score has previously been employed in a number of previous models designed to predict both short- [8] and long-term [23–26] outcomes in wider populations of patients undergoing thoracic surgery.

The risk model developed by Safi *et al.* (designed specifically to predict short-term mortality after pneumonectomy) contained 5 predictors (age, palliative indication for surgery, current alcohol use, raised preoperative leucocyte count and coronary artery disease), of which 2 were included in our multivariable analyses and none of which emerged as significantly associated with any of the primary outcomes. Unfortunately, the model was developed using statistically unreliable measures of calibration and internal validation [27, 28]. Moreover, the variables themselves are also somewhat unusual. For example, we do not support the use of pneumonectomy as part of palliative treatment for lung cancer. Our external validation suggests that the model is unsuitable for predicting in-hospital mortality after pneumonectomy.

The Thoracoscore model [29], designed to predict short-term mortality in patients undergoing all thoracic procedures, has been externally validated in a cohort of 243 patients undergoing pneumonectomy by Qadri *et al.* [30], the results of which demonstrated that measures of both discrimination and calibration were inadequate. The fact that no current model seems able to adequately risk stratify patients undergoing pneumonectomy is of particular concern, as this is a patient group where postoperative mortality and morbidity are exceptionally high, and life expectancy is frequently limited.

### Limitations

The main limitation of this study is its retrospective nature, which comes with drawbacks such as a higher rate of missing data in comparison to prospective studies. The length of time from which the data has been obtained (2012–2018) is also a potential limitation as thoracic surgical practice continues to evolve over time. However, limiting the study to a more recent time period would inevitably further reduce the sample size. This phenomenon was seen in the UK Pneumonectomy Outcome Study trial, which limited data collection to a single calendar year. Hence, although 28 UK centres participated in the trial, the total sample size was only slightly greater than the number of patients included in this study (*n* = 312).

## CONCLUSION

Undergoing pneumonectomy for primary lung cancer is associated with a high risk of mortality, extending far beyond the traditional perioperative period. Many studies have identified a wide range of risk factors associated with adverse outcomes, including, in this study, receiving neoadjuvant therapy (90-day mortality and 1-year mortality), Performance Status score (1-year mortality and overall survival) and advanced disease (overall survival). However, a lack of consensus regarding risk factors remains. Unfortunately, whilst the high rate of adverse outcomes associated with this operation means that accurate risk stratification is of particular importance, no model has as yet emerged as being acceptable for use. A national project to analyse outcomes and identify risk factors in this small but important cohort of lung cancer patients is required to ensure that appropriate tools are developed to aid and augment the decision-making process.

## SUPPLEMENTARY MATERIAL

Supplementary material is available at *ICVTS* online.

## ACKNOWLEDGEMENTS

We would like to acknowledge the members of the North West Thoracic Surgery Collaborative team who assisted with data collection: Leah Argus, Sarah Michael, Sabrina Mason, Dilraj Bhullar, Emmanuel Obale, and Nils Cristopher Fritsch. The surgeons who contributed to this study are Rajesh Shah, Piotr Krysiak, Kandadai Rammohan, Eustace Fontaine Felice Granato, Mark Jones, and Singh Soon (Manchester University NHS Foundation Trust) and Richard Page, Steven Woolley, Michael Shackcloth, Julius Assante-Siaw, Mike Poullis, and Neeraj Mediratta (Liverpool Heart and Chest Hospital).

## Funding

No funding was received for this work.

**Conflict of interest:** none declared.

## Data availability statement

All data files for this manuscript are held in the NCORR database. Data are available upon reasonable request.

## Author contributions

**Annemarie Brunswicker:** Formal analysis; Project administration; Writing—original draft; Writing—review & editing. **Marcus Taylor:** Conceptualization; Data curation; Formal analysis; Methodology; Writing—original draft; Writing—review & editing. **Stuart W. Grant:** Conceptualization; Methodology; Supervision; Writing—original draft; Writing—review & editing. **Udo Abah:** Data curation; Writing—review & editing. **Matthew Smith:** Data curation; Writing—review & editing. **Michael Shackcloth:** Writing—review & editing. **Felice Granato:** Writing—review & editing. **Rajesh Shah:** Supervision; Writing—review & editing. **Kandadai Rammohan:** Conceptualization; Supervision; Writing—review & editing.

## Reviewer information

Interactive CardioVascular and Thoracic Surgery thanks Frank A. Baciewicz Jr, Haruhisa Matsuguma and the other, anonymous reviewer(s) for their contribution to the peer review process of this article.

## Supplementary Material

ivab340_Supplementary_Materials

## ABBREVIATIONS

CI: Confidence interval
DLCO: Diffusion capacity of the lung for carbon monoxide
IQR: Interquartile range
NCORR: Northwest Clinical Outcomes Research Registry
O:E: Observed to expected
