# Supplementary material for: Pneumonectomy for primary lung cancer: contemporary outcomes, risk factors and model validation
Source: Interact Cardiovasc Thorac Surg. 2021 Dec 6;34(6):1054–61. doi: 10.1093/icvts/ivab340 (PMC9159428; doi:10.1093/icvts/ivab340)
Supplement: ivab340_Supplementary_Materials [file ivab340_supplementary_materials.docx]

**SUPPLEMENTARY MATERIALS**

Table 1. Univariable analysis for peri-operative mortality

| **VARIABLE** | **ALIVE AT DISCHARGE & 30 DAYS**  **(N=239)** | **DEAD AT DISCHARGE OR 30 DAYS**  **(N=17)** | **P VALUE** |
| --- | --- | --- | --- |
| Age (years) (mean ±SD^a^) | 64.9 (±9.5) | 69.1 (±8.1) | 0.079 |
| Male sex (%) | 57.3% (n=137) | 58.8% (n=10) | 0.904 |
| Neoadjuvant therapy (%) | 6.3% (n=15) | 17.6% (n=3) | 0.076 |
| PS^b^ (median ±IQR^c^) | 1.0 (±0.0-1.0) | 1.0 (±0.5-1.0) | 0.972 |
| % Predicted DLCO^d^ (mean ±SD) | 71.2% (±16.4%) | 70.4% (±20.3%) | 0.858 |
| Creatinine (median ±IQR) | 71.0 (±64.0-82.0) | 72.0 (±67.5-88.0) | 0.516 |
| Anaemia (%) | 34.7% (n=83) | 35.3% (n=6) | 0.962 |
| Smoking (%) | 87.0% (n=208) | 94.1% (n=16) | 0.393 |
| IHD^e^ (%) | 7.1% (n=17) | 11.8% (n=2) | 0.480 |
| Right-sided resection (%) | 31.0% (n=74) | 47.1% (n=8) | 0.169 |
| Advanced (stage III/IV) disease (%) | 42.3% (n=101) | 35.3% (n=6) | 0.574 |

Footnote a: standard deviation, b: performance status; c: interquartile range, d: diffusion capacity of the lung for carbon monoxide; e: ischaemic heart disease

Table 2. Univariable analysis for 90-day mortality

| **VARIABLE** | **ALIVE AT**  **90 DAYS**  **(N=231)** | **DEAD AT**  **90 DAYS**  **(N=25)** | **P VALUE** |
| --- | --- | --- | --- |
| Age (years) (mean ±SD^a^) | 65.0 (±9.6) | 67.0 (±7.8) | 0.312 |
| Male sex (%) | 56.3% (n=130) | 68.0% (n=17) | 0.260 |
| Neoadjuvant therapy (%) | 5.2% (n=12) | 24.0% (n=6) | <0.001 |
| PS^b^ (median ±IQR^c^) | 1.0 (±0.0-1.0) | 1.0 (±0.5-1.0) | 0.285 |
| % Predicted DLCO^d^ (mean ±SD) | 71.4% (±16.5%) | 68.8% (±18.8%) | 0.457 |
| Creatinine (median ±IQR) | 71.0 (±64.0-83.0) | 72.0 (±68.5-83.5) | 0.485 |
| Anaemia (%) | 34.6% (n=80) | 36.0% (n=9) | 0.891 |
| Smoking (%) | 87.4% (n=202) | 88.0% (n=22) | 0.937 |
| IHD^e^ (%) | 7.8% (n=18) | 4.0% (n=1) | 0.492 |
| Right-sided resection (%) | 30.3% (n=70) | 48.0% (n=12) | 0.072 |
| Advanced (stage III/IV) disease (%) | 43.7% (n=101) | 24.0% (n=6) | 0.058 |

Footnote a: standard deviation, b: performance status; c: interquartile range, d: diffusion capacity of the lung for carbon monoxide; e: ischaemic heart disease

Table 3. Univariable analysis for 1-year mortality

| **VARIABLE** | **ALIVE AT**  **1 YEAR**  **(N=184)** | **DEAD AT**  **1 YEAR**  **(N=72)** | **P VALUE** |
| --- | --- | --- | --- |
| Age (years) (mean ±SD^a^) | 64.8 (±9.6) | 66.1 (±8.9) | 0.326 |
| Male sex (%) | 57.1% (n=105) | 58.3% (n=42) | 0.854 |
| Neoadjuvant therapy (%) | 4.9% (n=9) | 12.5% (n=12) | 0.032 |
| PS^b^ (median ±IQR^c^) | 1.0 (±0.0-1.0) | 1.0 (±1.0-1.0) | 0.001 |
| % Predicted DLCO^d^ (mean ±SD) | 71.4% (±16.6%) | 70.3% (±17.0%) | 0.627 |
| Creatinine (median ±IQR) | 71.0 (±63.0-82.0) | 71.0 (±66.0-83.8) | 0.307 |
| Anaemia (%) | 30.4% (n=56) | 45.8% (n=33) | 0.020 |
| Smoking (%) | 87.5% (n=161) | 87.5% (n=63) | 1.000 |
| IHD^e^ (%) | 7.6% (n=14) | 6.9% (n=5) | 0.855 |
| Right-sided resection (%) | 29.9% (n=55) | 37.5% (n=27) | 0.241 |
| Advanced (stage III/IV) disease (%) | 38.6% (n=71) | 50.0% (n=36) | 0.096 |

Footnote a: standard deviation, b: performance status; c: interquartile range, d: diffusion capacity of the lung for carbon monoxide; e: ischaemic heart disease
